# Integrative Analysis of Metabolomic, Proteomic and Genomic Data to Reveal Functional Pathways and Candidate Genes for Drip Loss in Pigs

**DOI:** 10.3390/ijms17091426

**Published:** 2016-08-30

**Authors:** Julia Welzenbach, Christiane Neuhoff, Hanna Heidt, Mehmet Ulas Cinar, Christian Looft, Karl Schellander, Ernst Tholen, Christine Große-Brinkhaus

**Affiliations:** 1 Institute of Animal Science, University of Bonn, Endenicher Allee 15, 53115 Bonn, Germany; jwel@itw.uni-bonn.de (J.W.); cneu@itw.uni-bonn.de (C.N.); heidt@ibla.lu (H.H.); mucinar@erciyes.edu.tr (M.U.C.); cloo@itw.uni-bonn.de (C.L.); ksch@itw.uni-bonn.de (K.S.); etho@itw.uni-bonn.de (E.T.); 2 Institute for Organic Agriculture Luxembourg, Association sans but lucratif (A.S.B.L.), 13 Rue Gabriel Lippmann, L-5365 Munsbach, Luxembourg; 3 Department of Animal Science, Faculty of Agriculture, Erciyes University, Talas Bulvari No. 99, 38039 Kayseri, Turkey

**Keywords:** drip loss, pork quality, metabolomics, proteomics, enrichment analysis, genome-wide association study (GWAS), candidate genes

## Abstract

The aim of this study was to integrate multi omics data to characterize underlying functional pathways and candidate genes for drip loss in pigs. The consideration of different omics levels allows elucidating the black box of phenotype expression. Metabolite and protein profiling was applied in *Musculus longissimus dorsi* samples of 97 Duroc × Pietrain pigs. In total, 126 and 35 annotated metabolites and proteins were quantified, respectively. In addition, all animals were genotyped with the porcine 60 k Illumina beadchip. An enrichment analysis resulted in 10 pathways, amongst others, sphingolipid metabolism and glycolysis/gluconeogenesis, with significant influence on drip loss. Drip loss and 22 metabolic components were analyzed as intermediate phenotypes within a genome-wide association study (GWAS). We detected significantly associated genetic markers and candidate genes for drip loss and for most of the metabolic components. On chromosome 18, a region with promising candidate genes was identified based on SNPs associated with drip loss, the protein “phosphoglycerate mutase 2” and the metabolite glycine. We hypothesize that association studies based on intermediate phenotypes are able to provide comprehensive insights in the genetic variation of genes directly involved in the metabolism of performance traits. In this way, the analyses contribute to identify reliable candidate genes.

## 1. Introduction

Pork quality is the result of complex interactions between genetic and environmental effects like rearing and slaughtering conditions, and carcass and meat processing. One important commercially interesting pork quality parameter is the ability of meat to retain water, also known as water-holding capacity (WHC). In order to characterize WHC in pork, drip loss is measured. This fluid, mainly from muscle cells, resigns from the meat surface without any mechanical force other than gravity and is influenced by shrinkage of the myofibrils, pH value, and temperature post mortem (p.m.) [1,2]. Average drip loss in *Musculus longissimus dorsi* (LD) is around 1% to 5% [3]. Heritability estimates of WHC vary to a large extent between 0.01 and 0.31 [4]. This wide range could be explained by breed effects and large measurement errors of drip loss due to the multifactorial environmental effects [5]. Structural causes of drip loss concerning the muscle fibers and the biological processes associated with pork quality have been largely investigated and comprehended [6,7,8]. However, genetic mechanisms and interactions between different levels of metabolic regulation underlying drip loss are not fully understood [9,10,11].

Genetic studies, using standard approaches to identify candidate genes, already revealed several quantitative trait loci (QTL) and candidate genes for drip loss in pigs [11,12,13]. However, it can be expected that genome-wide association (GWA) studies based on functional, metabolic phenotypes or metabotypes reduce the risk to detect false-positive associations [14,15]. Several studies have demonstrated that the results of any single omics analysis, like an association analysis of SNPs and phenotypic expression as implemented by GWAS, may not be sufficient to decode extremely complex biological mechanisms [16]. In the case of multifactorial traits, metabotypes can be used in order to improve the accuracy of the phenotypic measurement. The combined analysis of different omics levels provides a promising tool to increase the information density between genome and phenotype. Thereby, integrative approaches for overall analysis of the entire cascade of genome and metabolic levels (transcriptome, metabolome and proteome) provide a potential prospective to identify reliable bio markers (transcripts, proteins, metabolites) and genetic markers (SNP, QTL, candidate genes) [17]. The knowledge of functional associated omic variables/markers including interactions between genetic and environmental factors may provide a comprehensive new insight into underlying biological processes in muscle growth and meat quality [16].

In recent years, the innovative technologies to record hundreds or thousands of omics profiles simultaneously and to analyze their relation to different traits were extensively developed further and established in many meat production sectors [18,19].

For meat scientists, the final objective is to identify meat quality genetic markers (like SNPs) or bio markers which are quantifiable on live animals or early p.m. on the carcass in order to orientate meat production towards the most adapted processes in meat processing or distribution circuits [16]. For this purpose, until now, a variety of genetic approaches was applied (see [11,12,13]). In the last decade, several scientific groups investigated different omics levels or integrated two or more omics levels to identify candidate transcripts or genes. For example, Te Pas et al. [20], Rohart et al. [21], Muroya et al. [22] and Welzenbach et al. [23] investigated the suitability of metabolite profiles and metabolic pathways in prediction of pork quality traits. Heidt et al. [24] applied a combined genomics and transcriptomics approach to reveal candidate genes for drip loss. The investigation of metabolic components, like metabolites and proteins, as new, more reliable phenotypes is a research focus in enhancement of meat quality traits [25]. D’Alessandro et al. [26] used a combined metabolomic and proteomic analysis to investigate the biochemical background of breed-specific meat quality differences. Apart from a few exceptions (see [27]), there have been very few studies combining more than two omics levels to identify candidate genes and QTL for pork quality, until now.

The aim of this study is the integration of omics levels genome, proteome and metabolome to elucidate underlying functional pathways and corresponding candidate genes for drip loss. Based on the increased information density due to the consideration of proteome and metabolome, we expect that our GWAS approaches based on metabolic traits contribute to identify true candidate genes with higher accuracy.

## 2. Results

In this study, metabolite and protein profiling, Enrichment analysis and GWAS were performed on 97 F_2_ Duroc × Pietrain (DuPi) pigs. The mean drip loss was 1.97%, with a minimum of 0.4% and a maximum of 5.3% (Table 1). In total, 1993 metabolites in each LD sample were quantified, using gas chromatography mass spectrometry (GC-MS) and liquid chromatography-quadrupole time of flight mass spectrometry (LC-QTOF/MS). However, out of these, only 128 metabolites were matched to their related Kyoto Encyclopedia of Genes and Genomes (KEGG) identifiers (IDs). According to the results of Neuhoff et al. [28], 40 proteins with expected significance for drip loss were quantified in the tissue samples. In the case of 35 proteins, we were able to annotate those with entrez gene identifiers. See Table S1 for drip loss phenotypes and profiles of the annotated metabolites and proteins.

### 2.1. Biological Pathways Involved in the Metabolite and Protein Abundance

In total, 163 metabolic components (128 metabolites and 35 proteins) were assigned to 219 KEGG pathways that potentially are involved in muscle to meat conversion and manifestation of meat quality characteristics. Based on the Wilcoxon rank sum test in 10 out of 219 KEGG pathways, the metabolites and proteins were significantly enriched (*p* ≤ 0.05) due to functional connectivity (Table 2). These pathways comprised in total 18 metabolites and four proteins and can be roughly distinguished into energy-relevant processes like “Glycolysis/gluconeogenesis” and “Pyruvate metabolism” and into pathways associated with different metabolic diseases like “Type II diabetes mellitus” and “NAFLD” (Non-alcoholic fatty liver disease). “Sphingolipid metabolism” (*p* = 0.014), that comprised four metabolites, was the most significantly enriched composition of metabolic components. Most metabolites and proteins were assigned to a single pathway. Of particular importance were across pathway components that might be indicators of key regulators with a strong impact on drip loss. As an example, the metabolites glucose and pyruvic acid are participants in six and five different pathways, respectively. The strongest overlapping induced by the metabolites glucose and pyruvic acid can be observed between glycolysis, methane and pyruvate metabolism showing that these pathways are closely linked. In contrast, the most significant pathway sphingolipid metabolism has only one link to the methane metabolism due to overlapping metabolite serine, whereas the remaining involved metabolites are exclusively members of sphingolipid metabolism.

Regarding the target trait drip loss, five metabolic components were significantly (*p* ≤ 0.05) correlated (Table 1). Metabolites pyruvic acid, methylglyoxal and glucosylceramide were significantly positive correlated while the proteins pyruvate kinase (muscle) (PKM) and triose phosphate isomerase 1 (TPI1) were negative correlated with drip loss. However, the correlation coefficient was not above a value of 0.22 in any case (Table 1).

### 2.2. Whole-Genome Association Analysis for Drip Loss and Metabolites and Proteins of Selected Biological Pathways

Beneath the meat quality trait drip loss itself, 22 metabotypes (18 metabolites and four proteins) were analyzed within a GWAS. In total, 44,844 SNPs were tested for association with at least one of the 22 metabolic traits or meat quality trait drip loss itself. In order to ensure the statistical power and accuracy of GWAS possible population stratification was considered [26]. In this context, principal components (PCs), which condensed the genetic relationship between animals, were considered in the statistical model as covariates. Depending on the investigated trait, between two and ten PCs were considered in order to avoid negative effects of population stratification on the validity of the GWAS analysis (Table 3). In most traits, the genomic inflation factor λ was close to one with a range of 1 to 1.05. Accordingly to the λ-thresholds (1.05) suggested by Price et al. [29] our correction was sufficient to remove disturbing population stratification. Only in the case of phosphoethanolamine the λ value (1.08) was slightly too high (Table 3).

Applying a moderate significance threshold with a false discovery rate (FDR) of *q* ≤ 0.10, the GWA studies revealed 871 (without double counting) significant associations for 15 traits, including drip loss, three proteins and 11 metabolites. These SNP were distributed over almost all porcine chromosomes. Four hundred thirty one SNPs showed a chromosome-wide significance levels of *q* ≤ 0.05 but no SNP was detected as genome-wide significant (*q* ≤ 0.01).

The average number of significant SNPs per trait was 66, with a minimum of two SNPs (for glucose) and a maximum of 249 SNPs for fructose-1,6-biphosphatase-2 (FBPase). The majority of the SNPs was significant at a moderate chromosome-wide level (*q* ≤ 0.1). The highest proportion of explained variance was observed for SNPs that affected glucosylceramide, dihydroxyacetone phosphate (glycerone-p) and d-glycerate-3-phosphate (DG3P). The most significant SNPs were detected for metabolite hydroxylpyruvic acid (*q* ≤ 2.19 × 10^−2^).

The average number of detected SNPs per chromosome is 67 and the highest numbers of significant SNPs were detected on *Sus scrofa* chromosome (SSC) 14, 17 and 18. For drip loss, we detected SNPs on SSC 16 and 18, which explain a maximum of variance proportion of 8.8%. Based on the distance to neighboring significant SNPs on the chromosome (1 Mb), we condensed the SNPs into 330 important QTL regions with an average of 29 QTL per chromosome (Table 3).

**Figure 1:**
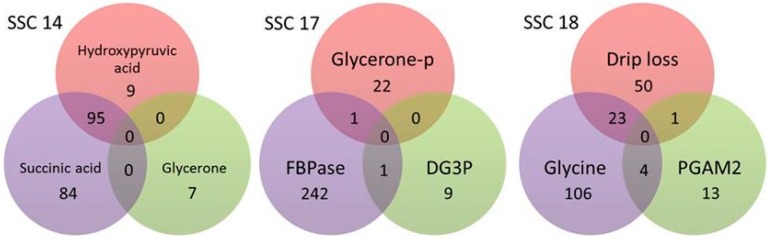
Overlapping SNPs at *Sus scrofa* chromosomes (SSC) 14, 17 and 18. GWAS procedures resulted in varying numbers of significant SNP (*q* ≤ 0.1) per trait. On some chromosomes there are overlapping SNPs with meaning for two traits. Drip loss measured in *Musculus longissimus dorsi* (LD) 24 h post-mortem (p.m.); Glycerone-p = dihydroxyacetone phosphate; PGAM2 = phosphoglycerate mutase 2 (muscle); FBPase = fructose-1,6-bisphosphatase 2; DG3P = d-glycerate-3-phosphate.

On several chromosomes we identified 126 (45) SNPs (QTL) which were significant for more than one trait. These SNPs are located on SSC 1, 7, 8, 14, 17 and 18. As presented in Figure 1 the most overlapping exists between metabolites hydroxypyruvic acid and succinic acid on SSC 14. Moreover, the overlapping on SSC 18 is of particular interest, because it indicates a metabolic process comprising glycine and phosphoglycerate mutase 2 (PGAM2) that influences drip loss (Figure 1). On SSC 7, there was only one overlapping SNP of glucose and fructose-6-phosphate (F6P). In contrast, on SSC 1 and 8, we indeed detected significant SNPs for two traits but the QTLs are located in distant chromosomal regions.

The functional annotation of the 871 significantly associated SNPs revealed 1430 genes that are located in a distance of ≤1 Mb to the SNPs and thereby are in linkage disequilibrium to our significant SNPs (Table 4). 257 SNPs are localized in an intron region of a specific gene. These genes, which are mainly located on SSC 14, 17 and 18, might be important potential candidate genes for drip loss and associated metabolic traits and processes (Table 4).

For the identification of potential candidate genes, we concentrated on the most important QTL regions with a high density of significant SNPs for different traits. These SNPs were selected based on the following three criteria: The SNPs had to be:

1. Chromosome-wide significant (at least *q* ≤ 0.1);
2. within the “Top 10” or “Top 25” of significant SNPs for metabolic traits or drip loss;
3. exonic or intronic.

Using these criteria we identified 23 potential candidate genes for drip loss and nine associated metabolic components (Table 5). SSC 18 is of particular interest, because on this chromosome we identified candidate genes for drip loss, glycine and PGAM2. The number of detected genes for a single trait varied between one and six. On SSC 4 six genes in a range of 20 Mb were detected for protein PKM. The importance of each candidate gene is indicated by one to five significant intronic SNPs. Five genes (*ZNHIT6*, *HLCS*, *ANK3*, *RASGEF1A* and *LRGUK*) harbour more than one intronic SNPs. Based on the QTL comprising five intronic SNPs in a small range of 0.29 Mb, it might be reasonably assumed that HLCS is a very promising candidate gene for FBPase. Most significant intronic SNPs with highest proportion of explained variance in a range of 15.28% to 17.44% were detected for glucosyl-ceramide, glycerone-p and glycine (Table 5).

**Figure 2:**
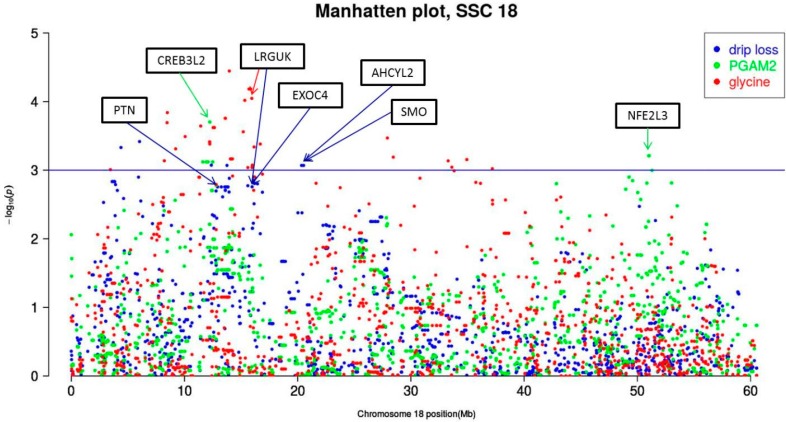
Chromosome-wide Manhattan plot of *Sus scrofa* chromosome (SSC) 18. Drip loss measured in Musculus longissimus dorsi (LD) 24 h post-mortem (p.m.); PGAM2 = phosphoglycerate mutase 2; the declaration of gene symbols (in black boxes) can be obtained from Ensembl or http://www.ncbi.nlm.nih.gov/genegenes.

For drip loss, five candidate genes were identified on SSC 18 (Table 5 and Table 6). The most significant SNPs (*Var*_max_ = 8.82%; *p*_min_ ≤ 6.58 × 10^−5^) associated with drip loss were detected on SSC 16, but these SNPs do not fulfill the previously described conditions to detect potential candidate genes (Table 6). Distributed over four regions, SSC 18 harbors two genes for PGAM2, four genes for drip loss and one gene (*LRGUK*) significantly associated with drip loss and glycine. Because *LRGUK* is in linkage disequilibrium with *EXOC4* that was associated with drip loss as well, this region ranging from 15.9 Mb to 16.1 Mb is of particular interest. From 12.2 Mb to 12.9 Mb there is a second interesting region with two candidate genes, for PGAM2 and drip loss, respectively. The Manhattan plot of SSC 18 is presented in Figure 2. Moreover, the Manhattan plots of SSC 1, 4, 6, 10, 13, 14 and 17 are shown in Figure S1.

## 3. Discussion

### 3.1. Systems Biological Approach or Integrated Analysis of Genome, Proteome and Metabolome to Elucidate the “Muscle to Meat” Black Box

Based on the multitude of possible post-transcriptional events, the genetic information flow from SNPs to phenotypic variations is not linearly dispersed in living organisms and samples collected p.m. [30]. This situation describes the black box between genes and phenotypes that needs to be opened to detect genetic variation influencing complex traits. Several studies have demonstrated that the results of single omics analysis, like standard GWAS procedure, may not be sufficient to decode extremely complex biological mechanisms [16]. A possible solution is to integrate different omics levels in genetic analyses and to analyze the entire cascade of metabolic levels. The omic levels proteome and metabolome were chosen for our analysis because we expected that these metabotypes are the final products of specific pathways and thereby are closely connected with classical target phenotypes routinely measured in animal production [20,21]. While the genome (SNP information) contains the information on which allele variants exist, the other omics levels indicate which genes are actually being expressed and which pathways are active. Therefore, metabolites and proteins constitute essential links between genetic information and phenotypical expression of complex traits and might be used in genetic association studies to improve the statistical power and to reveal less false positive, redundant results [21]. The concentration of metabolites and proteins in muscle and blood compared to drip loss is less influenced by environmental effects and thereby can be used as more accurate phenotype to identify candidate genes. This means, intermediate phenotypes might be more appropriate to estimate the genetic potential of the individuals than the performance trait itself. For example, a pig with excellent genetic potential for high meat quality and low drip loss might show high drip loss caused by bad environmental factors and management effects. In this case, drip loss is a poor indicator for the effective genetic potential of the individual.

To elucidate biological pathways affecting a trait, the consideration of the proteome is advantageous compared to the transcriptome. This can be assumed because the amount of proteins is not only regulated by a constant level of transcript expression but also by many possible genetic interacting mechanisms of protein regulation/modification and connected activation of other pathways [31]. In a similar context, Ala-Koperla et al. [32], Kadarmideen [33] and Widmann et al. [31] have stated that systems biological approaches are valuable and powerful in identifying key causal and highly predictive genetic variants for complex traits as well as in building up complex genetic regulatory networks.

### 3.2. Impact of Metabolic Pathways and Involved Metabolites and Proteins for Drip Loss

In this study, metabolite profiling was based on an untargeted metabolomics approach to uncover the whole metabolome. Compared to that, proteins were profiled more specific by means of a targeted proteomics approach using the absolute quantification of 40 proteins that have been shown as important indicators for drip loss in previous investigations. For the final enrichment analysis 128 annotated metabolites and 35 proteins were used. Five proteins were rejected because of missing entrez gene identifier. The drastic reduction of the number of metabolites from 1865 to only 128 is a severe bottleneck, so that it is highly probable that even metabolites with strong influence on drip loss were excluded. This situation is caused by the fragmentary information of biochemical functions of metabolites that is stored in metabolome databases. According to Chagoyen and Pazos [34], this lack of scientific fundamentals and principles of physiological and biochemical processes of higher life forms is a big challenge in systems biology studies. In a similar way, Chagoyen and Pazos [34] argued that there is a need of more accurate profiling tools for omic phenotypes in order to get a more comprehensive insight into the metabolic processes.

Our enrichment analysis considered all available annotated metabolome and proteome information and revealed 10 functional KEGG pathways with significant (*p* ≤ 0.05) enriched components. The applied test mean-rank gene-set enrichment (MR-GSE) statistic is based on Pearson’s correlation coefficients between metabotypes and drip loss and averages the ranks of the applied statistics instead of the statistics themselves. This procedure makes the results less influenced by individual components in the set of variables [35] and is the main difference to other usually applied testing procedures, like the Tktest of Tian et al. [36]. Further details are given by Ackermann and Strimmer [37].

In summary, it can be expected that the underlying function of our applied enrichment test has enough power to detect overrepresented groups of variables (e.g., genes or metabotypes), even if the effects are very small or the amount of data is not sufficient to detect the important variables individually [35]. This argument can be used to explain, why our enrichment analysis has resulted in functional sets of metabotypes although correlation coefficients between individual metabotypes and drip loss do not significantly deviate from zero (Table 1).

In our study, we observed particularly pathways and corresponding key regulators which affect muscle metabolism related to meat quality traits. Glycolysis, pyruvate and methane metabolism are strongly connected and belong to the most important energetic processes that influence the muscle to meat conversion [38,39]. Because drip loss strongly depends on p.m. energetic processes in muscle, the meaning of glycolysis and pyruvate metabolism is obvious. After slaughtering, in muscle tissues, anaerobe metabolic processes predominate and, in glycolysis, glycogen is released via glucose to pyruvic acid. Under aerobic conditions, pyruvic acid is metabolized in citrate cycle and oxidative phosphorylation [39]. In the case of stress before slaughtering, in hypoxic tissues the rate of oxidative processes like glycolysis is increased and pyruvic acid does not flow into glycolysis but is transferred to lactic acid. Accumulation of lactic acid goes along with pH decrease to 5.6 [40]. The meaning of metabolic processes associated with energy metabolism for drip loss is confirmed by a multitude of studies. Among others, Binke [41], Scheffler and Gerrard [39] and D’Alessandro et al. [26] allocated the relevance of glycolysis and pyruvate metabolism for meat quality. The coincidence of low early pH values and high temperature in muscle lead to partial denaturation of proteins and reduction of intercellular space. Thereby, lipids are dissolved from membranes, permeability of membranes is increased and drip loss is the result [6]. In cell exudate dissolved lipids clarify the connection between drip loss and activity of sphingolipid metabolism that includes the metabolization of ceramides, phosphoethanolamine and serines. The relation between drip loss and associated lipids and acids has been already described by Lambert et al. [42] and Poulsen et al. [43].

As a result of our enrichment analysis, the metabolite glycine is associated with drip loss. In methane metabolism the enzyme glyoxylate transaminase catalyzes the metabolization of metabolite glyoxylate into glycine or hydroxypyruvic acid (www.genome.jp). High glycine contents indicate a higher rate of glycolytic processes. A high glycolytic potential is known to be related with high drip loss. The link between drip loss and glycine was already described by Lim et al. [44], who observed higher drip loss in the case of higher glycine level in porcine skeletal muscle cells.

The meaning of PKM that is involved in pathways glycolysis/gluconeogenesis, pyruvate metabolism and type II diabetes mellitus (Table 2) was already clarified by several studies. For example, D’Alessandro et al. [26] confirmed that the PKM level appeared to be highly related to many meat quality criteria (WHC, meat color). Beneath PKM, PGAM2 and DG3P are also involved in glycolysis/gluconeogenesis and pyruvate metabolism. Under anaerobic conditions PGAM2 catalyzed the degradation of DG3P to 2-phosphoglycerates (Figure S2). Because high levels of glycolytic enzymes like phosphoglycerates are associated with increased drip loss [45], PGAM2 might be considered as an appropriate indicator for drip loss [46]. In addition, Davoli et al. [47] appreciated that the corresponding gene PGAM2, is a potential candidate gene for drip loss. The non-essential α-amino acid glycine is also product of catabolism of DG3P and is thus part of the same metabolic process as PGAM2.

Another section of glycolysis/gluconeogenesis illustrates the interactions of the enzymes FBPase and TPI1 and the metabolite dihydroxyacetone phosphate (glycerone-p). In gluconeogenesis FBPase converts fructose-1,6-biphosphate to F6P and in glycolysis phosphofructokinase catalyzes the metabolisation of F6P to fructose-1,6-bisphophate. In the following process of glycolysis, the enzyme fructose-bisphosphate aldolase converts fructose-1,6-bisphophate to glycerone-p. In the next step, glycerone-p is metabolized to glyceraldehyde-3-phosphate catalyzed by TPI1 (see Figure S2). Laville et al. [48] revealed a significant correlation between high TPI1 and tender meat with low drip loss. The meaning of FBPase for meat quality in pigs was described by Nam et al. [49]. They detected a lower FBPase expression in pigs with high drip loss and weak pH decrease p.m. [49].

Beneath metabolic processes whose activity directly depends on the individual energy resources, also sphingolipid metabolism is significantly associated with drip loss. With a *p*-value of 0.014, metabolic compounds in sphingolipid metabolism are the most strongly enriched metabolites and proteins in our study and thereby have an obvious effect on drip loss. According to Heidt et al. [24] there is a negative correlation between drip loss in DuPi pigs and transcripts associated with sphingolipid metabolism. According to our analysis, the metabolites ceramide, glucosylceramide, phosphoethanolamine and serine are involved in sphingolipid metabolism. Ceramides are lipid signaling molecules that activate proliferative or apoptotic pathways. They are products of the metabolism of free fatty acids to long-chain fatty acyl-CoAs (LCACoAs). LCACoAs can either be used for energy production through β-oxidation or undergo conversion to various signaling molecules, such as ceramide and diacylglycerol [50]. In the analysis of differentially expressed transcripts in DuPi pigs, Ponsuksili et al. [10] concluded that low drip loss is associated with ceramide pathways. Especially, drip loss is associated with ceramides as lipid signaling molecules that can activate proliferative or apoptotic pathways. The ceramide biosynthesis is part of the sphingolipid metabolism and ceramides arise from the conversion of complex sphingolipids such as glucosylceramides. According to Dobrowsky and Kolesnick [51], the levels of ceramides and glucosylceramides and the enzymes regulating their metabolism are associated with the cells response to stress. The degradation of membranes accompanies with cell stress and as a consequence drip loss has a relation to metabolites that indicate cell stress. This connection explains the relationship between drip loss and transformation products of sphingolipid metabolism.

The metabolic processes and their involved components and overlapping are presented in Figures S2–S5. Several metabolic components, such as glucose and pyruvic acid are involved in five of ten pathways relevant for drip loss. The connective position of these metabolites confirms their specific role as metabolic key players in the regulation of meat quality. The meaning of the disease related pathways (e.g., type II diabetes mellitus) and other processes (meiosis in yeast) for drip loss (Table 2) are based on the strong influence of specific involved metabolic components like glucose and pyruvic acid. It is not to be expected that there is in fact a physiological connection between meiosis in yeast and meat quality in pigs.

### 3.3. Significant Markers and Candidate Genes for Drip Loss and Associated Metabolic Traits

Drip loss is a complex trait that is genetically controlled by a variety of different genes [10] and is influenced by interaction of metabolic processes and participants like genes, transcripts, proteins and metabolites [6]. Against this background, it is problematic to identify genes with a strong influence on drip loss using classical GWAS approaches. Moreover, statistical problems like stratification within the investigated population increase the risk of false positive results. In order to adjust for population stratification we included PCs as fixed effects into the model of the GWAS procedures as suggested by Aulchenko et al. [52] and applied among others by Becker et al. [53] and Utsunomiya et al. [54]. Depending on the investigated trait (drip loss, protein, metabolite) the models contain 2 to 10 PCs, which lead to λ-values close to one. From these results we conclude a sufficient elimination of population stratification without unacceptable reduction of the genetic variation. 

Instead of a Bonferroni correction, that favors the occurrence of false negative associations [55], we used the q-value which based on the FDR to correct for multiple testing. Storey and Tibshirani [56] suggested including the FDR in GWAS to provide a better balance between statistical significance and power to detect true effects. As it has been recommended by Benjamini and Hochberg [57], we set a relaxed significant threshold of *q* ≤ 0.10.

**Figure 3:**
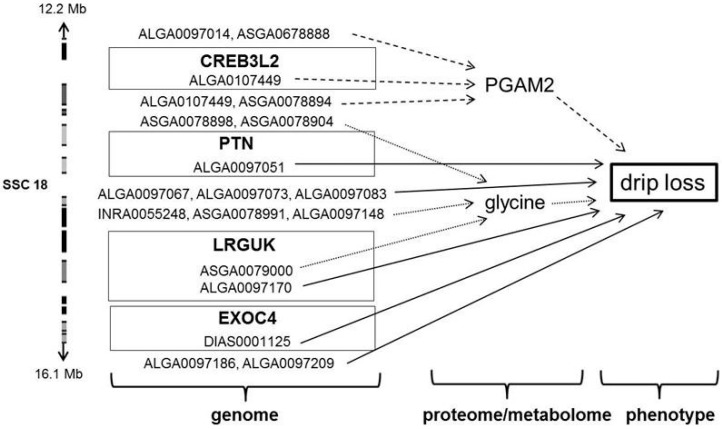
Region on *Sus scrofa* chromosome (SSC) 18 with potential candidate genes for drip loss and associated metabolic traits phosphoglycerate mutase 2 and glycine. Drip loss measured in *Musculus longissimus dorsi* (LD) 24 h post-mortem (p.m.); PGAM2 = phosphoglycerate mutase 2; fat solid arrows = direct relation between SNPs and drip loss; thin solid arrows = indirect relation between SNPs and drip loss via metabolite glycine; dotted arrow = indirect relation between SNPs and drip loss via protein PGAM2; genes in boxes: *CREB3L2 = cAMP responsive element binding protein 3 like 2*; *PTN = Sus scrofa pleiotropic factor beta*; *LRGUK = leucine-rich repeats and guanylate kinase domain containing*; *EXOC4 = exocyst complex component 4.*

The performed GWAS procedures resulted in a varying number of significant SNPs for drip, 11 metabolites and three proteins. The total of 871 significant SNPs are spread across the entire porcine genome, but concentrated on SSC 14, 17 and 18. For drip loss itself, promising candidate genes are located on SSC 18. This region has been earlier described by Jennen et al. [58] and Liu et al. [11]. In the region around 12 Mb, the meaning of “*Sus scrofa* pleiotropic factor beta” (*PTN*) (*q* ≤ 6.26 × 10^−2^) is highlighted by the direct neighborhood of gene “*cAMP responsive element binding protein*” (*CREB3L2*)*. CREB3L2* was identified by the GWAS of the protein PGAM2, which revealed an intronic SNP (ALGA0107449) as one of the most significant marker (Table 5). The family of cAMP response element binding proteins is crucial for a variety of cellular processes including cell proliferation, differentiation, apoptosis, extra-stimuli and stress response [59]. Although the meaning of *CREB3L2* so far was not precisely described for meat quality, our results suggest that this gene seems to have a relevant influence in energy metabolism in skeletal muscle that is indicated by its interacting effect on PGAM2, glycine and drip loss (Figure 3).

In the second interesting region on SSC 18 from 15.9 to 16.1 Mb, two intronic SNPs located in the gene “*Leucine-rich repeats and guanylate kinase domain containing” (LRGUK)* were found. These SNPs are ranked in the Top 10 list for drip loss as well as for glycine. The nearby gene “*Exocyst complex component 4*” (*EXOC4*) is also significantly associated with drip loss. *EXOC4* is part of the exocyst complex (Exo70), which is involved in insulin-stimulated glucose transport. Due to Laramie et al. [60], in humans polymorphisms near *EXOC4* and *LRGUK* on chromosome 7 are associated with type 2 diabetes and fasting glucose. The metabolic pathway that is regulated by the polymorphisms near *EXOC4* and *LRGUK* potentially is also relevant for drip loss in pork, because fasting glucose also effectsthe pH decrease in muscle p.m. and drip loss. The investigations of Leheska et al. [61] demonstrated that fasting before slaughtering yielded in a significant lower glucose level and weaker pH decrease in muscle p.m. and in less drip loss. In the third interesting region on SSC 18 around 20 Mb, directly next to each other genes “*Adenosylhomocysteinase-like 2*” (*AHCYL2*) and “*Smoothened, frizzled class receptor*” (*SMO*) are located and significantly associated with drip loss. Just like the polymorphism between *EXOC4* and *LRGUK*, *AHCYL2* is associated with type 2 diabetes [62]. Until now, there is no further evidence that this chromosomal region has an influence on meat quality. The effect of gene “*Nuclear factor, erythroid 2-like 3*” (*NFE2L3*) at 51 Mb, associated with protein PGAM2, fits into the same metabolic background like the previously described genes [63]. In summary, the multitude of significant SNPs detected for drip loss and associated metabotypes gives an ambiguous indication that in the described regions on SSC 18 promising candidate genes for drip loss can be expected.

In this study, the most significant SNPs were detected on SSC 1. Two SNPs (*p* ≤ 2.23 × 10^−5^ and *p* ≤ 1.59 × 10^−5^) associated with glycerone-p and glucosylceramide, are located within the genes “*Ectonucleotidepyrophosphatase/phosphodiesterase 3*” (*ENPP3*) and “*Sterile alpha motif domain containing 4a*” (*SAMD4A*). *ENPP3* is associated with lipid and fatty acid metabolism and it has been reported by to Liu et al. [64] that this gene affects fat deposition and skeletal muscle growth in pigs. *SAMD4A* is also associated with lipid metabolism [65] and influences the metabolisation of glucosylceramides that is part of sphingolipid metabolism. Combining biological knowledge found in literature and the highly significant results of our enrichment analysis leads to the conclusion that the sphingolipid metabolism is one of the most important metabolic pathways associated with drip loss.

Beneath glucosylceramides, phosphoethanolamines are also key players in sphingolipid metabolism. Two genes significantly associated with this metabolite were detected on SSC 6 (Table 5). “*Phosphatidylinositol 3-kinase, catalytic subunit type 3*” (*PIK3C3*) is involved in the regulation of hepatic glucose output, glycogen synthase, and antilipolysis in typical insulin target cells such as those in the liver, muscle and fat tissue [66]. Among others, *PIK3C3* influences the cellular response to glucose starvation (GO term: 0042149). This biological process describes the change in state or activity of a cell (in terms of movement, secretion, enzyme production, gene expression, etc.) as a result of deprivation of glucose. According to Kim et al. [66] and Hirose et al. [67] a polymorphism in *PIK3C3* is associated with body weight and carcass fat in Landrace and Duroc pigs.

Moreover, we identified potential candidate genes for several metabolic components involved in glycolysis/gluconeogenesis. The protein PKM is one of the most prominent members of these pathways. The activity of PKM is decreased in the case of low glucose availability in muscle that is positive correlated with anabolic cellular processes. During the conversion of muscle to meat, the metabolic processes change into the catabolic range and if glucose is used up very early, the PKM level is significantly associated with the aberrant glycolysis leading to PSE development [39]. In our analysis, it was shown that PKM is influenced by six candidate genes on SSC 4. In the chromosomal region of 139 Mb, genes “*Guanylate-binding protein 4*” (*GBP4*) and “*Protein kinase N2*” (*PKN2*) are located. Zhao et al. [68] have identified GBP4 as a significant QTL for lean meat content of pigs by comparing two divergent pig breeds with respect to carcass composition traits. Fontanesi et al. [69] have reported markers close to *PKN2* that were associated with back fat thickness. SSC 4 harbors two genes *(ZNHIT6, DDAH1)* within a region of 142–143 Mb which were significantly associated with average daily gain in Large White pigs [69]. These polymorphisms seem to have a strong impact on the metabolic rate and the deposition of skeletal muscle mass.

Two SNPs on SSC 17 give evidence that “*Protein tyrosine phosphatase, receptor type*” (*PTPRT*) and “*VAMP (vesicle-associated membrane protein)—associated protein B and C*” (*VAPB*) are candidate genes that affect the metabolite DG3P. The protein encoded by *PTPRT* is a signaling molecule that regulates a variety of cellular processes including cell growth, differentiation, mitotic cycle, and oncogenic transformation. In humans, *PTPRT* is strongly associated with high-fat diet-induced obesity and insulin resistance [70,71]. Moreover, in beef cattle, Tiziato et al. [72] identified *PTPRT* as candidate gene for shear force. With respect to the negative correlation between intramuscular fat content and shear force both studies came to homogeneous results. The importance of *PTPRT* is additionally indicated by the fact that the most important intronic SNP of *PTPRT* is an overlapping SNP that is also significantly associated with protein FBPase (Figure 1). DG3P and FBPase are strongly connected in glycolysis/gluconeogenesis and PTPRT might be a key player in regulation of glycolysis and thus a promising candidate gene for several meat quality traits.

### 3.4. Challenges and Perspectives

As it has been postulated by Fiehn et al. [73] and Krastanov [74], the development and performance of omics approaches have revolutionized the collection of biological data. Detection, quantification and annotation of hundreds of thousands of variables in tissue or blood samples presupposes enormous progress in chip technology, technical profiling/screening method, expansion of biological databases and handling of high dimensional data sets. From the statistical point of view, there are some unsolved questions, how to weight or to integrate the different omic levels in a statistical model. Genomic selection tools provide solution to weight large amount of SNP information in the case of a limited number of animals [75]. In a similar way “omics based selection” (OBS) methods try to weight genetic, transcriptional and metabolic information in an optimal manner. Under the condition of a successful weighting of metabotypes and the correct consideration of exogenous factors and the time point of profiling, OBS has the perspective to be an effective strategy in animal breeding, monitoring of state of health and supply status (e.g., nutritional metabolomics) and early disease detection (e.g., molecular epidemiology). Finally it should be mentioned that the profiling of metabotypes is non-invasive and may be performed in living organisms [76]. However, because of the complex interaction of genes, transcripts, proteins and metabolites these methods are conceptually very demanding and generally accepted methods are still missing. Moreover, beneath not standardized statistical methods to integrate omics data, the possibilities of metabolite and protein annotation are limited due to the fragmentary information of regarding databases. As a solution, network analyses might be valuable for the integration of multi omics data and the indirect annotation of unknown omics components based on the functional connectivity within a module of the network. A further difficulty is the dynamics of metabotypes in dependence of environmental effects and processing conditions. Biochemical processes response very quickly and dynamic to changes in exogenous factors. While the genetic information remains constant during the lifetime of an individual, the expression of transcripts, proteins and metabolites is very dynamic and regulated by a large number of factors. Thus, proteomic and metabolomic approaches can be viewed as recording of the metabolic status at a specific time point in a system of steady dynamic nature. Consequently, in estimation of performance traits the time point of metabolite and protein profiling has to take into account precisely.

## 4. Materials and Methods

### 4.1. Animals, Tissue Collection, Phenotyping

This study is based on 97 animals of a reciprocal DuPi crossbreed. The animals were selected from F_2_ families and based on their extreme high or low values of drip loss [24]. The animals were kept and performance tested under standardized conditions at the Frankenforst experimental farm of the University of Bonn from 2002 until 2007. Data recording and sample collection were conducted strictly in line with the German law on animal welfare. The entire experiment, including applied standard operating procedures, was approved by the veterinary and food inspection, Siegburg, Germany (No. 39600305-547/15). All animals were slaughtered at an average of 180.5 days (range 151–223 days) and average carcass weight of 86.5 kg (range 73.0–101.8 kg). The phenotypes were recorded in a commercial slaughterhouse according to the rules of German performance stations [77]. Further information can be found in Liu et al. [11] and Heidt et al. [24].

Sample collection was performed about 10 min p.m., immediately after exsanguination. Tissue samples were rapidly dissected, snap-frozen in liquid nitrogen and stored at −80 °C. Drip loss was measured in LD using the bag method of Honikel and Kim [40]. The samples from LD between 13th/14th rib (one chop per individual) with a thickness of 2.5–3.0 cm were collected 24 h p.m., weighed, and suspended in a plastic bag. After storage for 48 h at 4 °C, the samples were reweighed and drip loss were calculated as a percentage of weight loss based on the initial weight of a sample. In the tested animals drip loss ranged between 0.4% and 5.3%, whereby 49 pigs have drip loss values of lower 1.5% and the remaining 48 pigs show drip loss values of ≥1.5%.

### 4.2. Untargeted Metabolite Profiling

For metabolite profiling we choose an untargeted approach to screen the entire metabolome. The metabolite spectra in the LD samples of 97 DuPi pigs were measured by Metabolomic Discoveries GmbH (Potsdam, Germany; www.metabolomicdiscoveries.com) via gas GC-MS and LC-QTOF/MS. GC-MS and LC-QTOF/MS facilitate the identification and quantification of a few hundred metabolites in a single tissue sample. Chromatography followed by mass spectrometry has a relatively broad coverage of compound classes, including organic and amino acids, sugars, sugar alcohols, phosphorylated intermediates and lipophilic compounds. With the combination of both methods it is possible to detect metabolites in a range of 50–1700 Dalton, with a precision of 1–2 part per minute (ppm) and a solution of mass/Δmass = 40.000 (Report METABOLOMIC DISCOVERIES GmbH). For details on the LC-QTOF/MS method see Lisec et al. [78]. Metabolites were identified and annotated in comparison to Metabolomic Discoveries’ databases, which resort to Human Metabolome Database (HMDB, www.hmdb.ca), METLIN (www.metlin.scripps.edu/) and Lipid Maps (www.lipidmaps.org/). Annotation of metabolites was based on mass assignment, retention behavior and structure information. Metabolites, which could not be annotated, are characterized by their accurate mass and retention time. For details in metabolite quantification and annotation see Welzenbach et al. [22]. Only metabolites with known KEGG-ID were used for further analysis. Table S2 contains a list of all KEGG-annotated metabolites we used for further analysis. KEGG-IDs were obtained using R packages KEGGREST, biomaRt and AnnotationDbi of Bioconductor (https://www.bioconductor.org) based on HMDB-IDs.

### 4.3. Targeted Protein Profiling

For the protein quantification and annotation we applied a two-step procedure. In the first step, an untargeted proteome profiling approach via isotope-coded protein labeling (ICPL) was used to determine the whole proteome (holistic approach) in LD samples of 42 DuPi pigs selected based on their extreme phenotypes of drip loss. In the second step (validation step), a set of 40 selected proteins was quantified in the 97 DuPi pigs of this study (targeted protein profiling approach).

The ICPL procedure, which based on stable isotope labeling, combined with mass spectrometry has emerged as a powerful tool to identify and relatively quantify thousands of proteins within complex protein mixtures [79]. In contrast to traditional proteomics approaches e.g., by 2D-gel electrophoresis, ICPL technology shows highly accurate and reproducible quantification of proteins [80]. The ICPL approach resulted in 825 quantified proteins. The identification (annotation) of the quantified proteins was based on mass spectra and database query amongst others with the ICPL-Quant software.

Based on the holistic ICPL approach and literature research, 40 proteins with expected meaning for drip loss were selected. These proteins were validated via selected reaction monitoring (SRM) in the 97 DuPi pigs of this study. Using a triple quadrupole mass spectrometer, targeted SRM offers high selectivity, sensitivity and a wide dynamic range in the quantitative analysis of small molecules [81]. The ICPL and SRM analyses were performed by TOPLAP GmbH (Munich, Germany). For a more detailed description of the ICPL and SRM application in our samples, see Kellermann [79] and Gallien et al. [81].

Based on the available entrez gene ID or ensemble peptide ID, R packages KEGGREST, biomaRt and AnnotationDbi of Bioconductor (https://www.bioconductor.org) were used to identify the corresponding KEGG-IDs of the proteins. In Table S3, a list of all entrez gene ID- and peptide ID-annotated proteins is presented.

### 4.4. Genome Profiling

DNA was extracted from LD using a Genomic DNA Purification Kit (Fermentas Life Science, Thermo Fisher Scientific, Waltham, MA, USA). DNA concentration was measured using a NanoDrop 8000 spectrophotometer (Thermo Scientific, Wilmington, DE, USA) and concentration was adjusted to 100 ng/µL by using double-distilled RNase and DNase free water. Illumina bead array technology (Porcine SNP 60 K Bead Chip) was used for genotyping the samples (Illumina, Inc., San Diego, CA, USA) in accordance with the protocol for SNP Infinium HD assay (http://Illumina.com). DNA (200 ng) was used for genome-wide amplification and fragmentation. A quality score for each genotype was generated. Sample preparation and genotyping has been described by Heidt et al. [24].

### 4.5. Statistical Analysis

#### 4.5.1. Quality Control and Annotation of Genetic Data

Quality control was performed as implemented in R package GenABEL [52]. SNPs were excluded from further analysis under the following conditions: (a) Minor allele frequency (MAF) < 1%; (b) Call rate < 95% and (c) strong deviation from the Hardy-Weinberg-Equilibrium (*p* < 10^−3^). After checking the quality of the data, 97 animals and 44,844 SNPs remained in the data set. 

Pig Sscrofa 10.2 (International Swine Genome Sequencing Consortium) [82] was used to annotate all investigated SNPs. In order to detect biologically relevant genes being in linkage disequilibrium with significant associated SNPs, the R package biomaRt [83] was used. This procedure of functional annotation filtered genes in a distance of up to 1 Mb around the significant SNP regions. We chose this window, because in our assumption there is an association between SNP and potential candidate gene if the distance is ≤1 Mb.

#### 4.5.2. Metabolite and Protein Enrichment and Pathway Analysis

In order to investigate the overrepresentation of specific metabolite and protein sets in different KEGG pathways, an enrichment analysis was performed based on corresponding annotated metabolites and proteins and the target trait drip loss. For assignment of metabolites and proteins to relevant metabolic pathways, R package biomaRt was applied [83].

The enrichment analysis was performed as implemented in R package limma [76]. The underlying test procedure of limma, called MR-GSE, was developed by Michaud et al. [35] and refers to a Wilcoxon rank-sum test. The test statistic ranks the sets of metabolic components based on Pearson’s correlation coefficients between paired samples of metabolites/proteins and drip loss. The result is a list of ranked compositions containing a varying number of metabolites and proteins. It was assumed that significantly (*p* ≤ 0.05) enriched sets of metabolic components represent specific functional pathways that might be associated with muscle metabolism and meat quality traits. The procedure computes a *p*-value to test the hypothesis that a set of variables (metabolites and proteins) tends to be more highly ranked in terms of a given test statistic compared to randomly chosen variables. The calculated *p*-value indicates whether a set of variables is statistically independent that means that the variables are on average less or equally correlated than randomly chosen variables (H_0_ hypothesis), or whether a set of metabolites and proteins is enriched because of functional connectivity (H_1_ hypothesis). In the following step, metabolites and proteins of significant enriched functional pathways were analyzed in a GWAS.

#### 4.5.3. Genome-Wide Association (GWA) Analysis

The GWAS for pork quality parameter drip loss and metabolites/proteins of significant pathways was applied using the R package GenABEL [52]. The phenotypic traits (drip loss, metabolite/protein expression values) were corrected for “slaughter date” (SD) and “slaughter weight” (SW):

(1) $$y_{ijk}=\mu +SD_{j}+\beta_{s}SW_{i}+\beta_{g}g_{ik}+e_{ijk}$$

where *y_ij_* is the phenotype of the *i*-th individual. Fixed effect *SD* and as covariable *SW* with regression coefficient β*_s_* are implemented in the model. Genetic effects were estimated via a fixed covariable “genotype” (*g_ik_*) and corresponding regression coefficient (β*_g_*). The significance of each SNP was tested using a fast score test. In order to verify potential stratification in our F_2_ DuPi population, the inflation factor λ, which depends on the squared original test statistic of the *i*-th SNP ($T_{i}^{2}$) was calculated as

(2) $$\lambda =\frac{Median\left(T_{i}^{2}\right)}{0.4549}$$

Aulchenko et al. [52] and Price et al. [29] showed that an inflation factor λ in the range of 1.0 to 1.05 is an indicator of a sufficiently corrected population stratification which can be analyzed with an acceptable risk of false positive results. Preliminary results of our analysis showed that λ deviates slightly from this optimum. This implies that some population stratification exist within our F_2_ DuPi pigs. In order to correct for this problem, within the fast score test, PCs estimated from the genomic kinship (EIGENSTRAT) [29,52], were included as fixed covariables. The genomic kinship matrix was used to reveal the PCs reflecting the axes of genetic variation and describing the stratification of the populations involved in this study. The number of PCs used in this step is variable and depends on the ability to correct different levels of population stratifications. The number of PCs was increased stepwise from one to 10 PCs and the final number of PCs was chosen so that the inflation factor λ was nearest to 1.

The correction of phenotypes, the estimation of the PCs and the association analysis was performed with the function “egscore” as implemented in the R-package GenABEL. In order to reduce the risk of false-positive associations, the SNP significance tests were corrected for multiple testing based on the *q*-value calculation. This approach is a significance measurement based on the false discovery rate (FDR) concept [56]. We chose a significance threshold of *q* ≤ 0.1. The variance explained by the respective SNP was calculated using following formula:

(3) $$Var\;\left(\%\right)=\frac{\chi_{1df}^{2}}{\left(n-2+\chi_{1df}^{2}\right)}$$

where *χ*^2^ is the result of the score test as implemented in GenABEL and n the number of individuals. This formula resulted from the transformation of a Student’s *t*-distribution into a *z*-distribution [84]. Based on a similar MAF, a similar allele substitution effect and a similar proportion of explained variance we assumed that SNPs within a distance of <1 Mb to each other belong to one QTL.

## 5. Conclusions

Systems biological approaches utilize the information content of all available omic variables (SNPs, transcripts, proteins, and metabolites) in order to clarify the physiological, biochemical and genetic background of complex traits. Theoretically, across omics utilization is advantageous in comparison to classical genetic approaches, which merely investigate associations between SNPs and phenotype.

In this context, Picard et al. [16] and van der Sijde et al. [85] have stated that there is an increasing interest to combine all the omic levels in a holistic omics approach to investigate the complexity of the molecular events beyond expected biological functions and to identify important genes. It can be expected that meat quality traits are influenced by a high number of interacting genes that are unknown or involved in unexpected functions, so that the across omics level approach used in our study is particularly useful. Based on the described integrated analysis of the omic levels genome, proteome, metabolome and phenotype, we increased the information density between genes and trait of interest to decode the complex biological mechanisms influencing drip loss and to reveal promising candidate genes. At least some of these genes have not been detected based on a standard GWAS procedure. The most promising candidate genes were located on SSC 18 where we detected several, partly overlapping QTL for drip loss itself and the intermediate phenotypes PGAM2 and glycine. These candidate genes need further investigations to identify underlying functional mutations affecting drip loss and the related metabolic processes.

Based on the results of this study, it was possible to confirm the already known findings about the importance of energy related metabolic processes influencing meat quality and particularly drip loss. On the other hand, this study also provides novel insights into the underlying biochemical pathways of drip loss. According to our findings, the sphingolipid metabolism is of particular importance for drip loss manifestation. The involved metabolites glucosylceramide and phosphoethanolamine are promising intermediate phenotypes for drip loss and revealed promising candidate genes on SSC 1. It can be expected that such integrated omics approaches might be successfully applied to clarify the biochemical and genetic background also in more complex traits than meat quality. Against this background, our study may be considered as a model investigation to test one possible procedure to combine different omic levels.

## Figures and Tables

**Table 1:** Descriptive statistics and phenotypic correlations between drip loss and metabotypes.

Traits	Mean ± SD ^1^	Min ^2^	Max ^3^	Correlation to Drip Loss ^4^
drip loss, %	1.97 ± 1.40	0.40	5.30	1
pH1	6.53 ± 0.22	5.89	6.94	−0.31 **
pH24	5.52 ± 1.12	5.32	6.06	−0.35 ***
**PKM**	26,454.10 ± 17,829.55	13.47	88,251.64	−0.20 *
**PGAM2**	5600.37 ± 4985.98	−10.77	32,935.16	−0.19
**FBPase**	27,407.08 ± 20,231.70	809.35	114,192.30	−0.11
**TPI1**	1754.68 ± 1526.65	32.13	7802.84	−0.21 *
pyruvic acid	4.32 × 10^−2^ ± 3.62 × 10^−2^	6.16 × 10^−3^	2.11 × 10^−1^	0.22 *
lactic acid	6.49 × 10^−1^ ± 3.28 × 10^−1^	1.88 × 10^−1^	1.64	0.08
glucose	9.02 × 10^−3^ ± 1.32 × 10^−2^	1.21 × 10^−4^	8.41 × 10^−2^	0.19
phosphoenol pyruvate	5.59 × 10^−2^ ± 8.95 × 10^−2^	1.80 × 10^−3^	0.53	0.13
glycerone-p	1.86 ± 1.10	2.48 × 10^−1^	5.85	0.07
DG3P	2.56 × 10^−1^ ± 4.09 × 10^−1^	2.61 × 10^−3^	2.61	0.14
fumaric acid	2.67 × 10^−3^ ± 1.25 × 10^−3^	5.50 × 10^−4^	7.23 × 10^−3^	0.12
succinic acid	1.38 × 10^−2^ ± 5.02 × 10^−3^	3.23 × 10^−3^	3.23 × 10^−2^	−0.02
malic acid	6.03 × 10^−3^ ± 2.92 × 10^−3^	8.85 × 10^−4^	1.64 × 10^−2^	0.11
methylglyoxal	9.62 × 10^−3^ ± 5.44 × 10^−3^	2.61 × 10^−4^	2.89 × 10^−2^	0.22 *
glycine	8.59 × 10^−2^ ± 2.39 × 10^−2^	4.84 × 10^−2^	1.62 × 10^−1^	0.11
hydroxypyruvic acid	1.06 × 10^−2^ ± 6.81 × 10^−3^	1.76 × 10^−3^	4.98 × 10^−2^	0.02
F6P	2.17 × 10^−2^ ± 3.43 × 10^−2^	2.91 × 10^−4^	2.25 × 10^−1^	0.12
serine	6.04 × 10^−3^ ± 2.99 × 10^−3^	1.76 × 10^−3^	2.15 × 10^−2^	−0.01
glycerone	1.41 × 10^−1^ ± 8.36 × 10^−2^	2.17 × 10^−2^	4.37 × 10^−1^	0.20
ceramide	1.68 × 10^−4^ ± 1.24 × 10^−3^	2.33 × 10^−6^	6.59 × 10^−4^	0.05
glucosylceramide	2.46 × 10^−3^ ± 4.72 × 10^−3^	1.69 × 10^−4^	2.72 × 10^−2^	0.21 *
phosphoethanolamine	8.57 × 10^−4^ ± 5.01 × 10^−4^	2.28 × 10^−4^	3.52 × 10^−3^	0.12

Drip loss measured in *Musculus longissimus dorsi* (LD) 24 h post-mortem (p.m.); pH measured 1 h post-mortem (p.m.) (pH1) and 24 h p.m. (pH24) in LD; ^1^ mean and standard deviation (SD); ^2^ minimum (Min); ^3^ maximum (Max); ^4^ calculation of correlation coefficients based on residuals; Mean, SD, Min and Max of proteins are based on signal dependent intensities of ion fragments (in mass) relative to time, so-called selection reaction monitoring (SRM) intensities; The units of the metabolite profiles are based on mass intensities, recorded by GC-MS and LC-QTOF/MS, normalized to an internal standard; * *p* ≤ 0.05, ** *p* ≤ 0.01, *** *p* ≤ 0.001; glycerone-p = dihydroxyacetone phosphate; PGAM2 = phosphoglycerate mutase 2 (muscle); PKM = pyruvate kinase (muscle); FBPase = fructose-1,6-bisphosphatase 2; TPI1 = triose phosphate isomerase 1; DG3P = d-glycerate-3-phosphate; F6P = fructose-6-phosphate; bold: proteins.

**Table 2:** Significant KEGG pathways for drip loss.

Pathway	KEGG-ID	*p*-Value *	Involved Metabolites and Proteins
Sphingolipid metabolism	00600	0.014	ceramide, glucosylceramide, phosphoethanolamine, serine
Type II diabetes mellitus	04930	0.018	pyruvic acid, glucose, **PKM**
Methane metabolism	00680	0.020	glycine, pyruvic acid, hydroxypyruvic acid, F6P, malic acid, serine, phosphoenol pyruvate, glycerone-p, glycerone, DG3P
Renal cell carcinoma	05211	0.027	fumaric acid, malic acid
Insulin secretion	04911	0.043	pyruvic acid, glucose
Meiosis yeast	04113	0.045	glucose
NAFLD	04932	0.045	glucose
Glycolysis/Gluconeogenesis	00010	0.045	pyruvic acid, lactic acid, glucose, phosphoenol pyruvate, glycerone-p, DG3P, **FBPase**, **TPI1**, **PKM**, **PGAM2**
Pyruvate metabolism	00620	0.053	fumaric acid, pyruvic acid, succinic acid, lactic acid, malic acid, phosphoenol pyruvate, methylglyoxal, **PKM**
Steptomycin biosynthesis	00521	0.056	glucose, myo-inositol

The enrichment analysis was performed based on 129 metabolites and 35 proteins. Overrepresentation of metabolic pathways defined by the KEGG database regarding to drip loss was tested using Wilcoxon’s rank sum test; * The pathway was considered significant if *p* ≤ 0.05; Kyoto Encyclopaedia of Genes and Genomes (KEGG)-ID = KEGG pathway ID; NAFLD = Non-alcoholic Fatty liver disease; glycerone-p = dihydroxyacetone phosphate; PGAM2 = phosphoglycerate mutase 2 (muscle); PKM = pyruvate kinase (muscle); FBPase = fructose-1,6-bisphosphatase 2; TPI1 = triosephosphate isomerase 1; DG3P = d-glycerate-3-phosphate; F6P = fructose-6-phosphate; bold: proteins.

**Table 3:** Results of association analyses.

Trait	ID	PC ^1^	λ ^2^	Number of Significant SNP/QTL per Porcine Chromosome ^3^													∑SNP ^4^	Min *p*-Value ^5^	Min *q*-Value ^6^	Max $\sigma_{y}^{2}$ ^7^
				1	2	3	4	6	7	8	10	13	14	16	17	18				
drip loss	-	10	1.007											4/4		74/20	78 ^#^	6.58	6.26	8.8
**PKM**	100158154	10	1				33/13										33 ^#^	10.7	7.84	14.3
**PGAM2**	100188980	10	1.06													18/7	18 ^#^	19.9	8.67	13.9
**FBPase**	100134828	10	1									5/1			244/92		118 *, 131 ^#^	1.98	2.27	16.6
glucose	C00031	10	1						2/2								2 ^#^	5.86	8.80	15.3
glycerone-p	C00111	10	1.046	4/1						7/4					23/10		34 ^#^	2.35	5.07	17.3
DG3P	C00197	10	1												10/5		2 *, 8 ^#^	1.50	2.19	17.3
succinic acid	C00042	2	1.03										179/64				122 *, 57 ^#^	29.3	5.07	13.3
glycine	C00037	10	1.05		97/41						2/2					133/48	102 *, 130 ^#^	3.39	4.67	17.1
hydroxyl-pyruvic acid	C00168	10	1										104/28				76 *, 28 ^#^	3.44	1.88	16.1
F6P	C00085	10	1						12/9								12 ^#^	8.00	7.69	14.8
glycerone	C00184	10	1										7/4				7 ^#^	7.95	8.56	14.8
ceramide	C00195	4	1.006							20/8							20 ^#^	11.8	8.02	14.4
glucosyl-ceramide	C01190	10	1.012	3/3		1/1											4 ^#^	1.59	6.64	17.4
phosphor-ethanolamine	C00346	10	1.08					15/8									11 *, 4 ^#^	15.4	3.81	14.5
∑SNP/QTL excluding double counting				7/4	97/41	1/1	33/13	15/8	13/10	27/12	2/2	5/1	195/80	4/4	275/100	197/54				
∑overlapping SNP/QTL ^8^									1/1				95/16		2/7	28/21				

Drip loss measured in Musculus longissimus dorsi (LD) 24 h post-mortem (p.m.) (%); ^1^ number of principal components (PCs) considered in genome-wide association (GWA) studies; ^2^ λ = inflation factor; ^3^ number of chromosome-wide significant associated SNPs and QTL per traits and chromosome (at least *q* ≤ 0.1); ^4^ sum of significant associated SNPs per traits (* *q* ≤ 0.05; ^#^ *q* ≤ 0.1); ^5^ minimal empirical *p*-value (times 10^−5^); ^6^ minimal *q*-value (times 10^−5^), based in the false discovery rate (FDR) concept; ^7^ maximal proportion of explained variance (%); ^8^ sum of overlapping SNP/QTL with meaning for two traits; ID = Entrez gene ID for proteins or KEGG compound ID for metabolites; glycerone-p = dihydroxyacetone phosphate; PGAM2 = phosphoglycerate mutase 2 (muscle); PKM = pyruvate kinase (muscle); FBPase = fructose-1,6-bisphosphatase 2; DG3P = d-glycerate-3-phosphate; F6P = fructose-6-phosphate; bold: proteins.

**Table 4:** Functional annotation of significant SNPs associated with drip loss and metabolic traits.

SSC ^1^	1	2	3	4	6	7	8	10	13	14	16	17	18	∑
Genes ^2^	30	148	4	65	31	48	70	15	12	375	13	367	252	1430
SNP ^3^	2/7	30/97	-/1	15/33	2/15	-/13	2/27	1/2	5/5	83/195	-/4	54/275	63/197	257/871

^1^ *Sus scrofa* chromosomes; ^2^ number of genes that are located in a distance of ≤1 Mb to the significant SNPs revealed by GWAS; ^3^ number of intronic SNPs in relation to the total number of significant SNPs per chromosome (without double counting of overlapping SNPs).

**Table 5:** Annotation of most promising SNPs for drip loss and associated metabolic components.

SSC ^1^	Trait	Gene ^2^	SNP ^3^	Position ^4^	Mut ^5^	MAF ^6^	eEff (se) ^7^		Chi2	Emp. *p*-Value ^8^	*q*-Value ^9^	*Var* ^10^
1	glycerone-p	*ENPP3*	INRA0001633	35387799	G/A	0.47	−4.00 × 10^−2^	(1.00 × 10^−2^)	18.68	0.22	5.07	17.35
	glucosylceramide	*SAMD4A*	ALGA0007238	204522804	C/A	0.47	−9.32 × 10^−5^	(2.15 × 10^−5^)	18.80	0.16	6.64	17.44
4	**PKM**	*NTNG1*	INRA0016801	123080603	G/A	0.27	−9.21 × 10^2^	(2.57 × 10^2^)	12.88	3.32	7.84	13.21
		*GBP4*	ASGA0023322	139599066	G/A	0.38	−6.43 × 10^2^	(1.77 × 10^2^)	13.26	2.71	7.84	12.72
		*PKN2*	M1GA0006779	139861416	C/A	0.43	6.76 × 10^2^	(1.89 × 10^2^)	12.88	3.32	7.84	12.40
		*ZNHIT6*	ALGA0029718	142789911	A/G	0.46	8.52 × 10^2^	(2.20 × 10^2^)	15.01	1.07	7.84	14.29
			ALGA0029732	142739989	G/A	0.39	9.23 × 10^2^	(2.49 × 10^2^)	13.70	2.14	7.84	13.09
			ALGA0029741	142730172	G/A	0.46	8.13 × 10^2^	(2.15 × 10^2^)	14.20	1.64	7.84	13.50
		*DDAH1*	ASGA0023626	143204232	A/G	0.40	9.05 × 10^2^	(2.43 × 10^2^)	13.86	1.97	7.84	13.21
		*WDR63*	INRA0018033	143449789	A/G	0.40	9.05 × 10^2^	(2.43 × 10^2^)	13.86	1.97	7.84	10.77
6	phosphor-ethanolamine	*PIK3C3*	DRGA0006746	118055075	G/A	0.26	2.91 × 10^−5^	(7.54 × 10^−6^)	14.93	1.76	3.81	14.36
		*TTLL5*	INRA0022204	120225026	C/A	0.26	2.91 × 10^−5^	(7.54 × 10^−6^)	14.93	1.76	3.81	14.36
10	glycine	*AKT3*	MARC0098464	18065301	C/A	0.34	−1.55 × 10^−3^	(3.80 × 10^−4^)	16.56	0.69	5.11	15.69
13	**FBPase**	*HLCS*	MARC0019610	210504370	G/A	0.49	6.54 × 10^2^	(1.70 × 10^2^)	14.71	1.25	8.64	13.92
			MARC0005075	210516458	A/C	0.49	6.54 × 10^2^	(1.70 × 10^2^)	14.71	1.25	8.64	13.92
			ASGA0089689	210516937	G/A	0.49	6.54 × 10^2^	(1.70 × 10^2^)	14.71	1.25	8.64	13.92
			ASGA0089950	210531047	A/G	0.49	6.54 × 10^2^	(1.70 × 10^2^)	14.71	1.25	8.64	13.92
			ASGA0097399	210534054	G/C	0.49	6.54 × 10^2^	(1.70 × 10^2^)	14.71	1.25	8.64	13.92
14	succinic acid	*ANK3*	MARC0033238	68550413	G/A	0.52	1.69 × 10^−4^	(4.59 × 10^−5^)	13.60	2.93	2.82	13.26
			ASGA0064107	68604989	A/G	0.52	1.69 × 10^−4^	(4.59 × 10^−5^)	13.60	2.93	2.82	13.26
		*RASGEF1A*	ALGA0078235	66284845	G/A	0.52	1.69 × 10^−4^	(4.59 × 10^−4^)	13.60	2.93	2.82	13.26
			ALGA0078240	66320818	A/C	0.52	1.69 × 10^−4^	(4.59 × 10^−5^)	13.60	2.93	2.82	13.26
			ALGA0078243	66332408	G/A	0.52	1.69 × 10^−4^	(4.59 × 10^−5^)	13.60	2.93	2.82	13.26
17	DG3P	*PTPRT*	MARC0016232	50694545	A/G	0.41	−1.96 × 10^−2^	(5.27 × 10^−3^)	13.88	1.94	6.53	13.49
		*VAPB*	H3GA0049968	65818274	A/G	0.48	1.71 × 10^−2^	(4.79 × 10^−3^)	12.78	3.51	6.53	12.55
18	PGAM2	*CREB3L2*	ALGA0107449	12234417	G/A	0.41	1.88 × 10^2^	(4.90 × 10^1^)	14.79	1.99	8.67	13.98
	drip loss	*PTN*	ALGA0097051	12921061	A/G	0.25	−7.81 × 10^−2^	(2.49 × 10^−2^)	9.87	17.6	6.26	5.61
	glycine	*LRGUK*	ASGA0079000	15942579	A/G	0.31	−1.63 × 10^−3^	(4.07 × 10^−4^)	16.06	0.90	1.66	15.28
	drip loss		ALGA0097170	15969549	G/A	0.45	−4.34 × 10^−2^	(1.38 × 10^−2^)	9.87	17.5	6.26	5.61
		*EXOC4*	DIAS0001125	16179365	G/A	0.48	4.15 × 10^−2^	(1.31 × 10^−2^)	10.08	15.6	6.26	5.72
		*AHCYL2*	H3GA0050495	20338092	A/G	0.28	−7.16 × 10^−2^	(2.14 × 10^−2^)	11.21	8.54	6.26	6.32
		*SMO*	ASGA0079098	20520014	G/A	0.30	−7.16 × 10^−2^	(2.14 × 10^−2^)	11.21	8.54	6.26	6.32
	**PGAM2**	*NFE2L3*	ASGA0100894	51012467	C/A	0.42	1.89 × 10^2^	(5.35 × 10^1^)	12.53	6.18	8.67	12.10

The SNP order complies with number of chromosomes and position on the chromosome; Selection of promising SNPs based on the criteria, that they are (1) chromosome-wide significant (at least *p* < 0.1); (2) within the “Top 10” significant SNPs per metabolic trait or “Top 25” for drip loss and (3) located within an annotated gene; ^1^ *Sus scrofa* chromosomes (SSC); ^2^ The declaration of gene symbols can be obtained from Ensembl or http://www.ncbi.nlm.nih.gov/gene; ^3^ None of the SNPs is located in an exon region of the regarding candidate gene; ^4^ position in BP (base pairs); ^5^ mutation (Mut); ^6^ minor allele frequency (MAF); ^7^ eEff = substitution effect and se = standard error; ^8^ empirical *p*-value, times 10^−4^; ^9^ *q*-value (based on the false discovery rate (FDR) concept), times 10^−2^; ^10^ Var = proportion of the explained variation [%]; glycerone-p = dihydroxyacetone phosphate; PGAM2 = phosphoglycerate mutase 2 (muscle); PKM = pyruvate kinase (muscle); FBPase = fructose-1,6-bisphosphatase 2; DG3P = d-glycerate-3-phosphate; bold: proteins.

**Table 6:** “Top 25” significant SNPs identified for drip loss and potential candidate genes.

	SNP	SSC ^1^	Position ^2^	Mut ^3^	MAF ^4^	eEff (se) ^5^		Chi2	Emp. *p*-Value ^6^	*q*-Value ^7^	*Var* ^8^	Located within a Gene ^9^
1	ALGA0089069	16	11629284	C/A	0.08	2.26 × 10^−1^	(5.65 × 10^−2^)	16.05	6.58 × 10^−5^	7.02 × 10^−2^	8.82	×
2	CASI0008411	16	23115634	G/A	0.10	1.89 × 10^−1^	(4.86 × 10^−2^)	15.03	1.12 × 10^−4^	7.02 × 10^−2^	8.30	×
3	MARC0097282	16	10946289	G/A	0.33	7.45 × 10^−2^	(1.95 × 10^−2^)	14.65	1.38 × 10^−4^	7.02 × 10^−2^	8.15	×
4	ASGA0072217	16	9183890	A/G	0.34	7.25 × 10^−2^	(1.93 × 10^−2^)	14.16	1.78 × 10^−4^	7.02 × 10^−2^	7.90	×
5	ALGA0111681	18	6026724	G/A	0.15	1.35 × 10^−1^	(3.78 × 10^−2^)	12.71	3.83 × 10^−4^	6.26 × 10^−2^	7.11	×
6	ASGA0104044	18	4388048	A/C	0.15	1.27 × 10^−1^	(3.61 × 10^−2^)	12.34	4.68 × 10^−4^	6.26 × 10^−2^	6.92	×
7	MARC0003904	18	12368984	G/A	0.35	−6.37 × 10^−2^	(1.90 × 10^−2^)	11.23	8.45 × 10^−4^	6.26 × 10^−2^	6.33	×
8	ASGA0078921	18	13751595	G/A	0.29	−7.46 × 10^−2^	(2.23 × 10^−2^)	11.21	8.53 × 10^−4^	6.26 × 10^−2^	6.33	×
9	H3GA0050495	18	20338092	G/A	0.30	−7.16 × 10^−2^	(2.14 × 10^−2^)	11.21	8.54 × 10^−4^	6.26 × 10^−2^	6.33	*AHCYL2*
10	ASGA0079098	18	20520014	A/G	0.30	−7.16 × 10^−2^	(2.14 × 10^−2^)	11.21	8.54 × 10^−4^	6.26 × 10^−2^	6.33	*SMO*
11	ALGA0105391	18	5935981	G/A	0.31	6.69 × 10^−2^	(2.07 × 10^−2^)	10.48	1.26 × 10^−3^	6.26 × 10^−2^	5.94	×
12	INRA0055248	18	13959002	G/A	0.47	−4.24 × 10^−2^	(1.31 × 10^−2^)	10.40	1.32 × 10^−3^	6.26 × 10^−2^	5.90	×
13	MARC0036783	18	16113241	A/G	0.47	−4.23 × 10^−2^	(1.32 × 10^−2^)	10.27	1.41 × 10^−3^	6.26 × 10^−2^	5.82	×
14	ASGA0098607	18	3614625	A/G	0.38	−5.35 × 10^−2^	(1.68 × 10^−2^)	10.20	1.47 × 10^−3^	6.26 × 10^−2^	5.79	×
15	ALGA0104874	18	3620895	A/G	0.38	−5.35 × 10^−2^	(1.68 × 10^−2^)	10.20	1.47 × 10^−3^	6.26 × 10^−2^	5.79	×
16	ASGA0088995	18	3741888	G/G	0.38	−5.35 × 10^−2^	(1.68 × 10^−2^)	10.20	1.47 × 10^−3^	6.26 × 10^−2^	5.79	×
17	H3GA0050278	18	3808173	A/G	0.38	−5.35 × 10^−2^	(1.68 × 10^−2^)	10.20	1.47 × 10^−3^	6.26 × 10^−2^	5.79	×
18	ASGA0078689	18	3833808	G/A	0.38	−5.35 × 10^−2^	(1.68 × 10^−2^)	10.20	1.47 × 10^−3^	6.26 × 10^−2^	5.79	×
19	DIAS0001125	18	16179365	G/A	0.48	4.15 × 10^−2^	(1.31 × 10^−2^)	10.08	1.56 × 10^−3^	6.26 × 10^−2^	5.72	*EXOC4*
20	ALGA0097186	18	16444813	G/A	0.47	4.20 × 10^−2^	(1.32 × 10^−2^)	10.06	1.58 × 10^−3^	6.26 × 10^−2^	5.71	×
21	ALGA0096804	18	3907848	G/A	032	5.58 × 10^−2^	(1.77 × 10^−2^)	10.01	1.63 × 10^−3^	6.26 × 10^−2^	5.69	×
22	ALGA0116114	18	15594213	A/G	0.46	−4.22 × 10^−2^	(1.34 × 10^−2^)	9.94	1.69 × 10^−3^	6.26 × 10^−2^	5.65	×
23	ALGA0097170	18	15969549	G/A	0.45	−4.34 × 10^−2^	(1.38 × 10^−2^)	9.88	1.75 × 10^−3^	6.26 × 10^−2^	5.62	*LRGUK*
24	ALGA0097051	18	12921061	A/G	0.25	−7.81 × 10^−2^	(2.49 × 10^−2^)	9.87	1.76 × 10^−3^	6.26 × 10^−2^	5.61	*PTN*
25	ALGA0097067	18	13674866	A/G	0.25	−7.81 × 10^−2^	(2.49 × 10^−2^)	9.87	1.76 × 10^−3^	6.26 × 10^−2^	5.61	×

The SNP order complies with raising *p*-value; ^1^ *Sus scrofa* chromosomes (SSC); ^2^ position in base pairs (BP); ^3^ mutation (Mut); ^4^ minor allele frequency (MAF); ^5^ substitution effect and standard error (se); ^6^ empirical *p*-value and significant thresholds, ^7^ *q*-value (based in the false discovery rate (FDR) concept); ^8^ Var = proportion of the explained variation [%]; ^9^ The declaration of gene symbols can be obtained from Ensembl or http://www.ncbi.nlm.nih.gov/gene, “×”, SNP is not located within a gene, none of the SNPs is located in an exon region of the regarding gene.

## Supplementary Materials

Supplementary materials can be found at www.mdpi.com/1422-0067/17/9/1426/s1.
