# Supplementary material for: Integrative Analysis of Metabolomic, Proteomic and Genomic Data to Reveal Functional Pathways and Candidate Genes for Drip Loss in Pigs
Source: Int J Mol Sci. 2016 Aug 30;17(9):1426. doi: 10.3390/ijms17091426 (PMC5037705; doi:10.3390/ijms17091426)
Supplement: Supplementary file 1 [file ijms-17-01426-s001.zip › ijms-140271-supple-final.pdf]

Supplementary Materials: Integrative Analysis of Metabolomic, Proteomic and Genomic Data to Reveal Functional Pathways and Candidate Genes for Drip Loss in Pigs

Julia Welzenbach, Christiane Neuhoff, Hanna Heidt, Mehmet Ulas Cinar, Christian Looft, Karl Schellander, Ernst Tholen and Christine Große-Brinkhaus

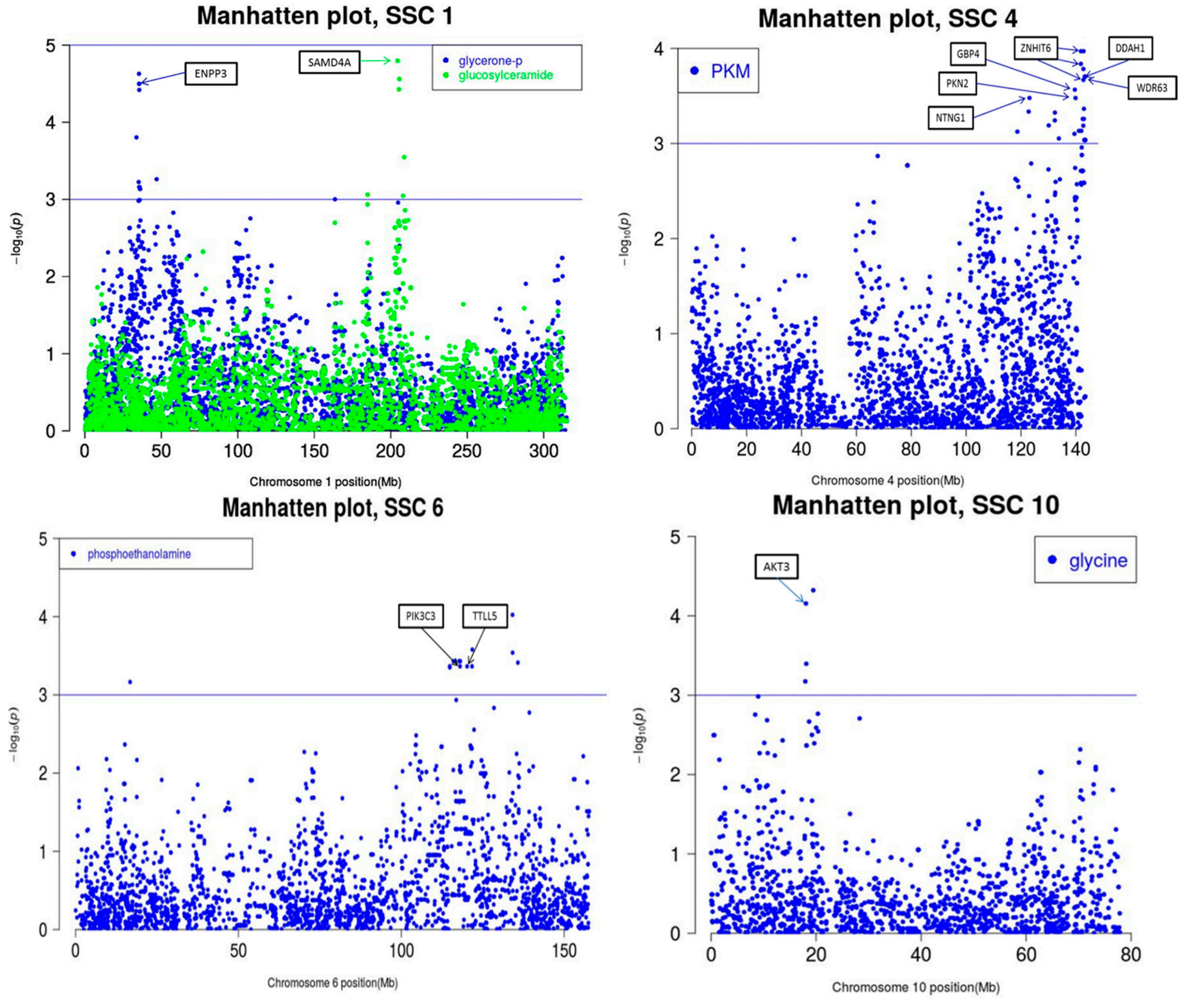

Figure S1. *Cont.*

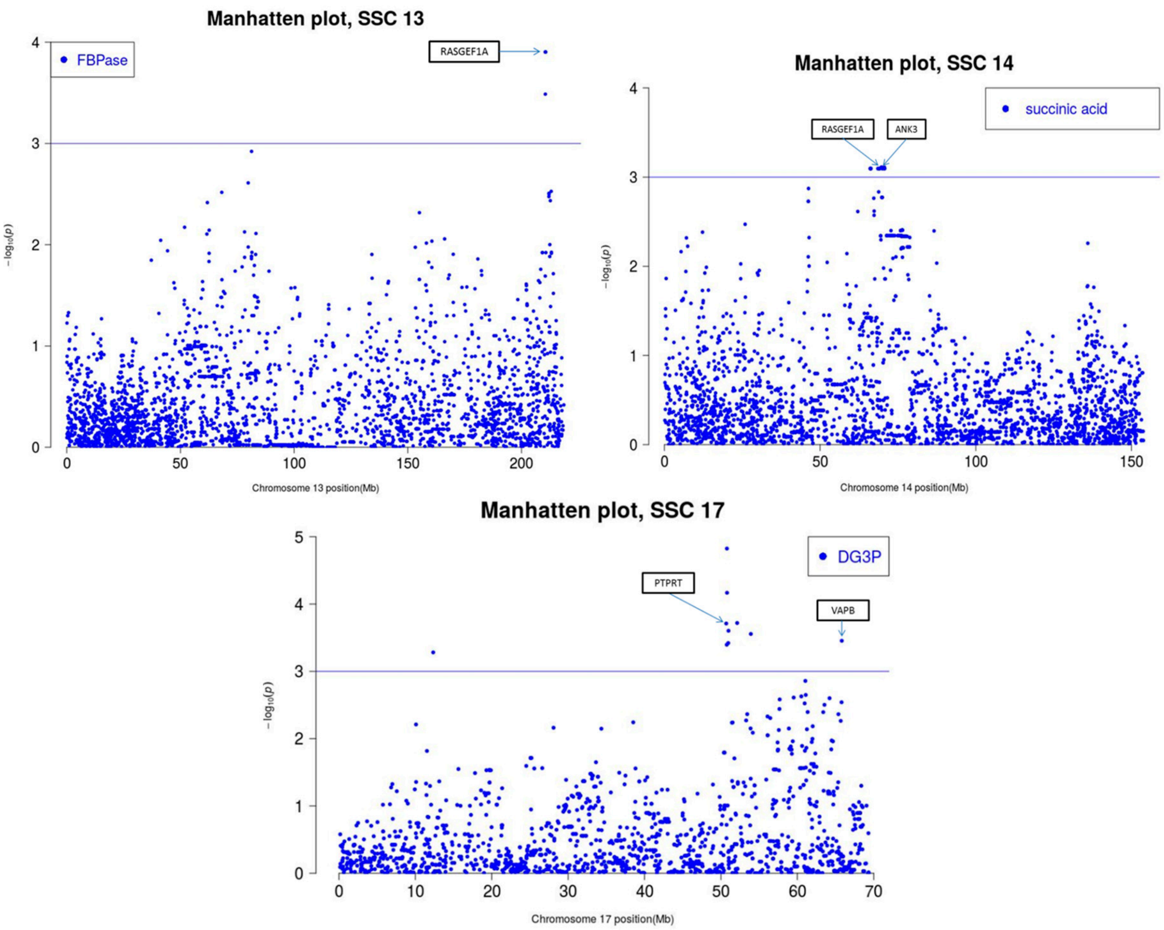

Figure S1. Chromosome-wide Manhattan plots of sus scrofa chromosomes (SSC) 1, 4, 6, 10, 13, 14 and 17. Glycerone-p = dihydroxyacetone phosphate; DG3P = D-glycerate-3-phosphate; PKM = pyruvate kinase (muscle); FBPase = fructose-1,6-bisphosphatase 2; the declaration of gene symbols (in black lettering) can be obtained from Ensembl or http://www.ncbi.nlm.nih.gov/genegenes.

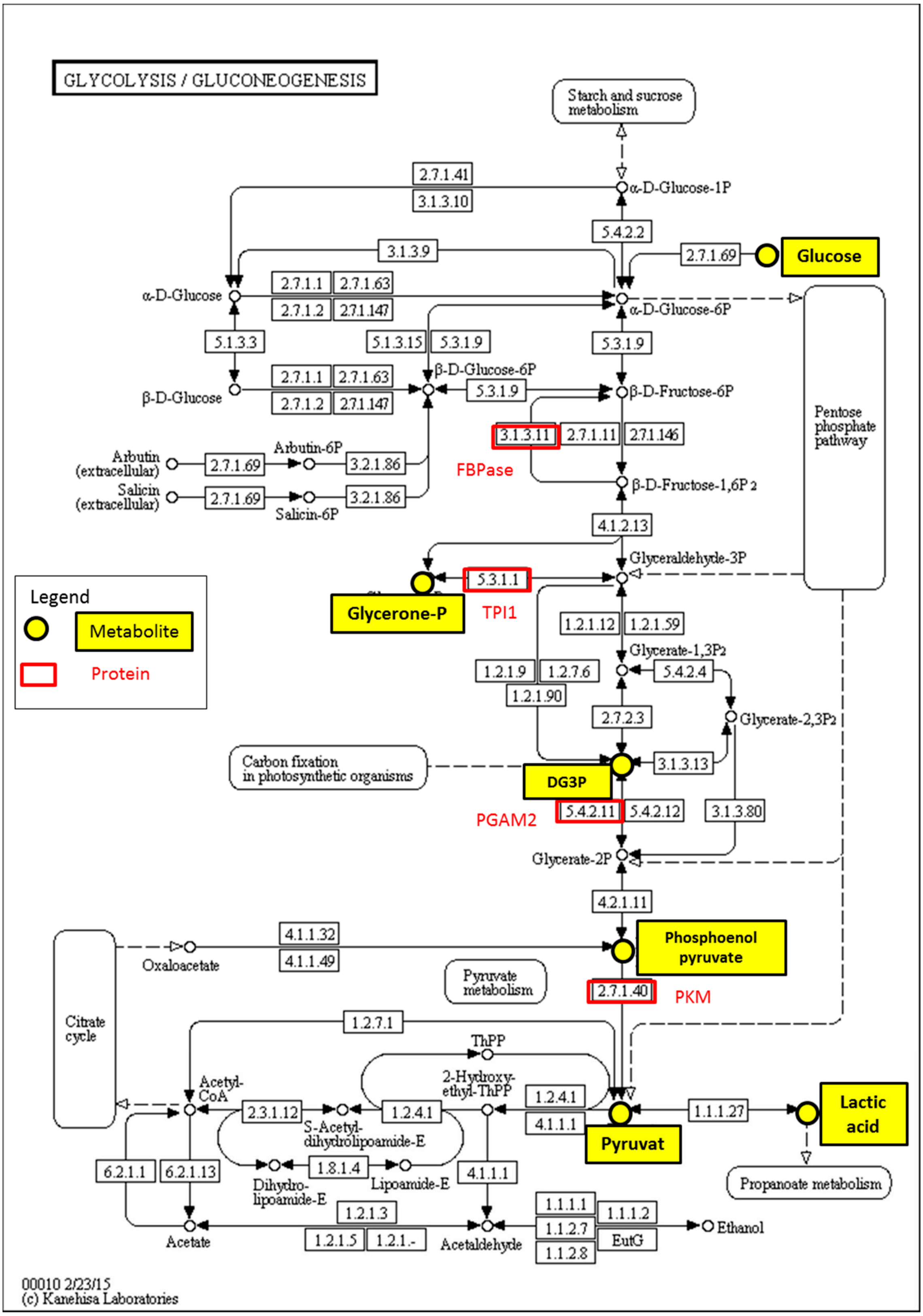

Figure S2. KEGG pathway "Glycolysis/Glyconeogenesis" (map00010). Metabolites and proteins revealed through the enrichment analysis are illustrated in yellow and red; Glycerone-p = dihydroxyacetone phosphate; PGAM2 = phosphoglycerate mutase 2 (muscle); PKM = pyruvate kinase (muscle); FBPase = fructose-1,6-bisphosphatase 2; TPI1 = triosephosphate isomerase 1; DG3P = D-glycerate-3-phosphate; The declaration of the other metabolites and proteins codes (in boxes) can be obtained from Kyoto Encyclopedia of Genes and Genomes (KEGG)-database (http://www.genome.jp/kegg/).

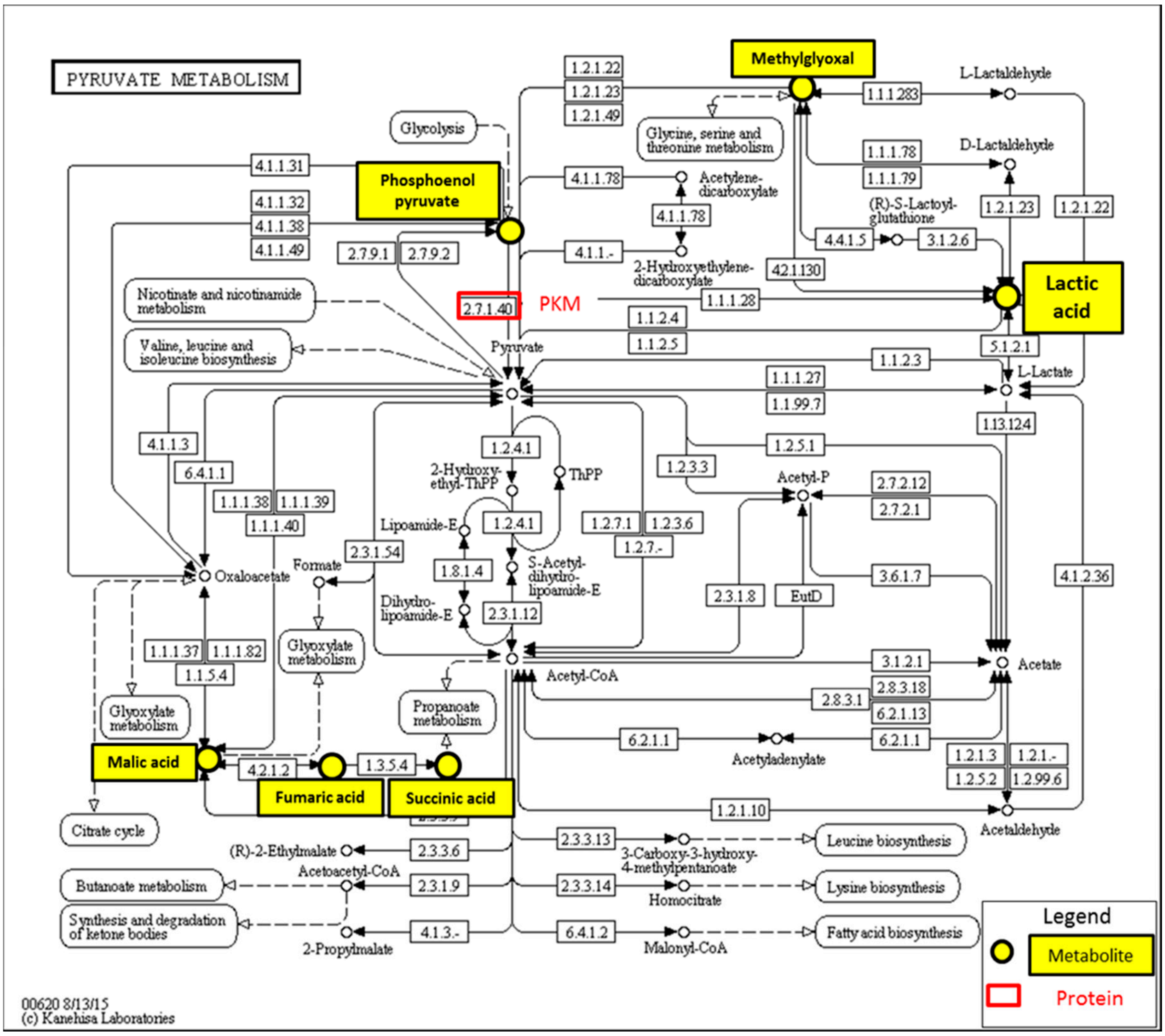

Figure S3. KEGG pathway "Pyruvate metabolism" (map00620). Metabolites and proteins revealed through the enrichment analysis are illustrated in yellow and red; PKM = pyruvate kinase (muscle); the declaration of the other metabolites and proteins codes (in boxes) can be obtained from Kyoto Encyclopedia of Genes and Genomes (KEGG)-database (http://www.genome.jp/kegg/).

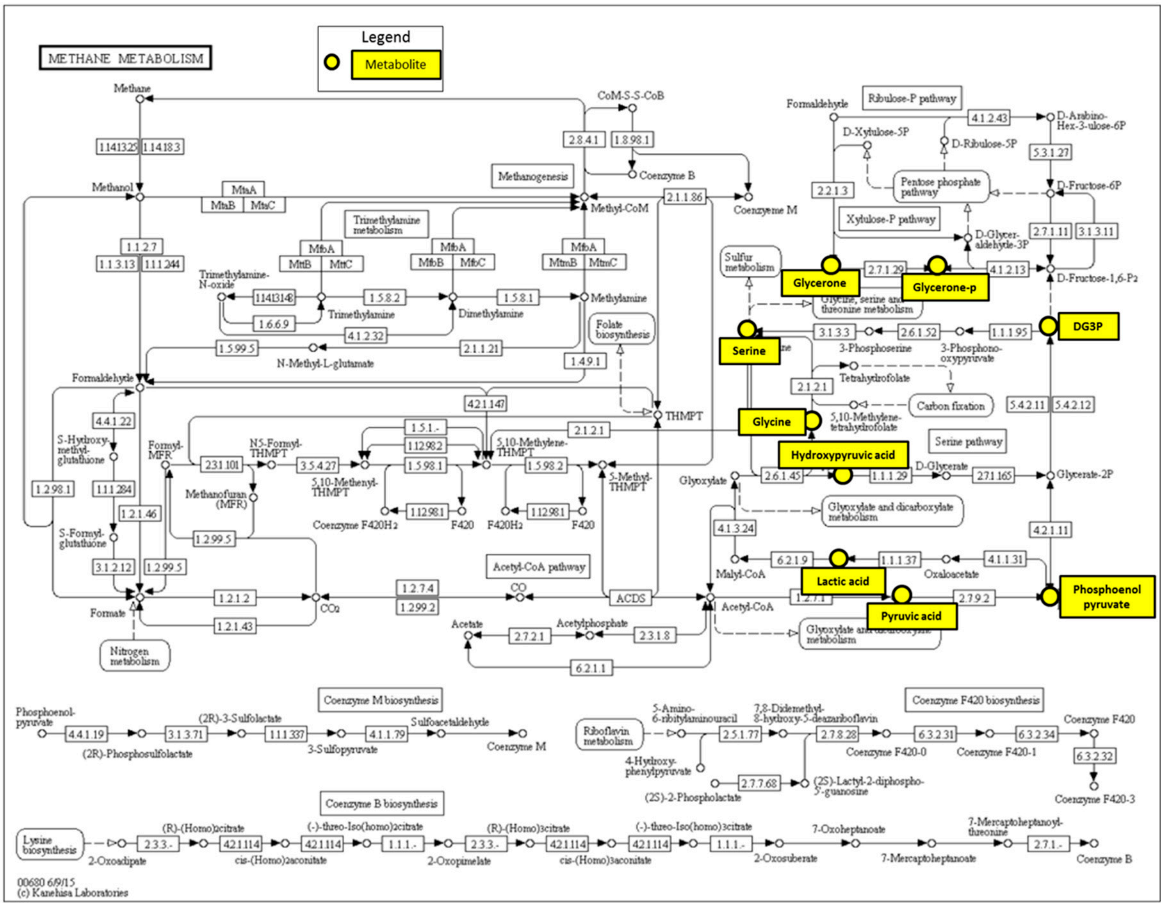

Figure S4. KEGG pathway "Methane metabolism" (map00680). Metabolites and proteins revealed through the enrichment analysis are illustrated in yellow and red; Glycerone-p = dihydroxyacetone phosphate; DG3P = D-glycerate-3-phosphate. The declaration of the other metabolites and proteins codes (in boxes) can be obtained from Kyoto Encyclopedia of Genes and Genomes (KEGG)-database (http://www.genome.jp/kegg/).

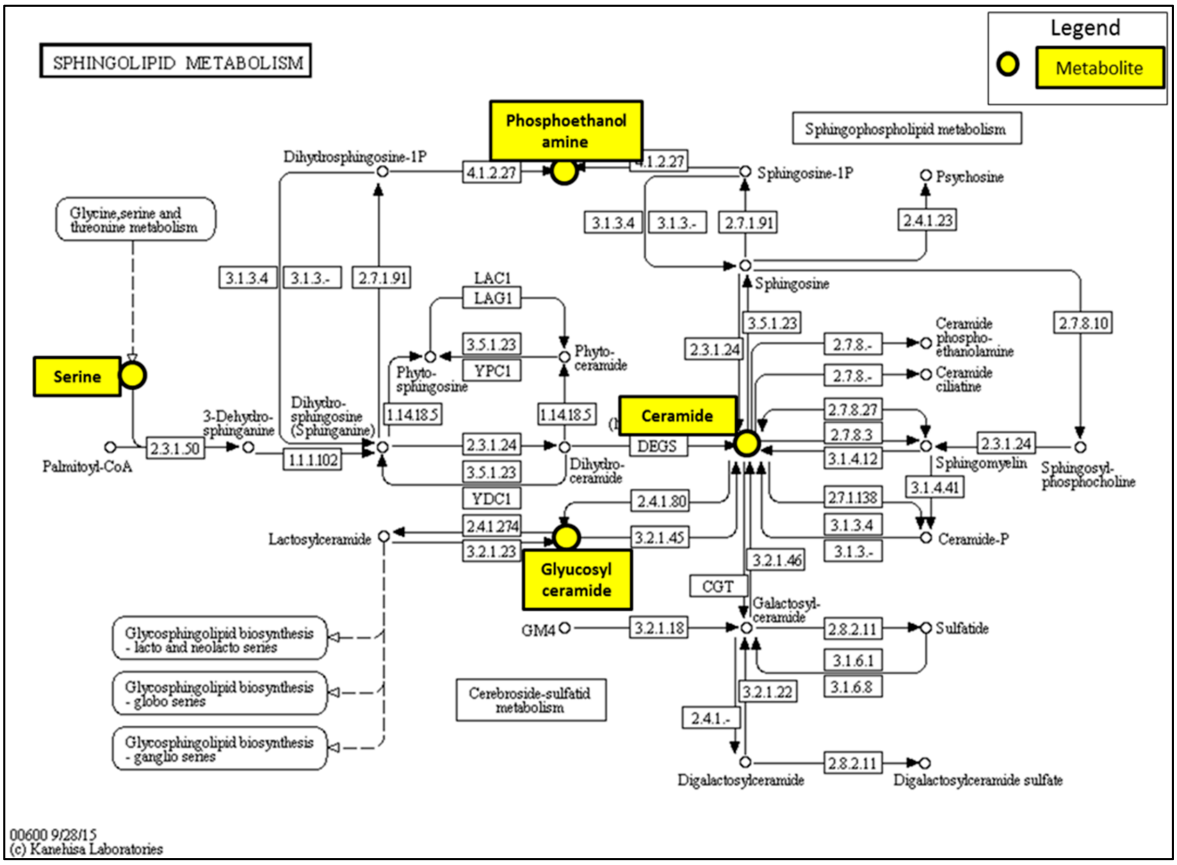

Figure S5. KEGG pathway "Sphingolipid metabolism" (map00600). Metabolites and proteins revealed through the enrichment analysis are illustrated in yellow and red; the declaration of the other metabolites and proteins codes (in boxes) can be obtained from Kyoto Encyclopedia of Genes and Genomes (KEGG)-database (http://www.genome.jp/kegg/).
